# An In‐Depth Review of the Genetics of the Non‐Classical HLA Class I Gene *HLA‐E* and Its Effects on Haematopoietic Cell Transplant Outcomes

**DOI:** 10.1111/tan.70344

**Published:** 2025-08-05

**Authors:** J. A. M. Lucas, S. G. E. Marsh, N. P. Mayor

**Affiliations:** ^1^ Anthony Nolan Research Institute, Royal Free Hospital London UK; ^2^ UCL, Cancer Institute, Royal Free Campus London UK

**Keywords:** diversity, genetics, haematopoietic cell transplant, *HLA‐E*, non‐classical HLA

## Abstract

*HLA‐E* is a non‐classical HLA class I gene with limited reported genetic variability and few published studies into full‐gene sequencing or population allele frequencies. Two protein variants, HLA‐E*01:01 and HLA‐E*01:03, are very common, accounting for 94%–100% of observed alleles in most studies performed to date. Frequently utilised exon‐based sequencing strategies have led to the assumption of *HLA‐E* being a near bi‐allelic gene; however, recent full‐gene sequencing studies have shown a greater degree of genetic variability than initially imagined. We carried out a literature review of *HLA‐E* genotype and ethnicity data, which suggested HLA‐E*01:03 is more common in Asian and, in particular, East Asian populations. Furthermore, HLA‐E*01:03:02 is more frequently observed than HLA‐E*01:03:01 in European and American populations, whereas HLA‐E*01:03:01 is found at higher frequencies in Asian populations. It has been proposed that HLA‐E may have a role in Haematopoietic Cell Transplantation (HCT) due to its interaction with NK and CD8^+^ T cells and its non‐canonical peptide binding repertoire. Here we also review published literature into the effects of *HLA‐E* genetics on HCT outcomes. Heterogeneity between cohorts muddies the waters; hence, studies report confounding effects of *HLA‐E* genotype and matching on HCT outcomes. The need for further *HLA‐E* sequencing of larger cohorts is evident to gain useful insight into the true genetic variability of *HLA‐E* and its impact on HCT.

## 1 Introduction

The human Major Histocompatibility Complex (MHC) is a region on the short arm of chromosome 6 (6p21.3) containing several hundred genes [1]. These genes are imperative to the body's immune system in mounting immunological responses against pathogenic threats. Arguably, the most important gene system in the MHC is HLA, with the encoded proteins of HLA class I being expressed on almost all nucleated cells of the body [2, 3]. HLA molecules present “self” and foreign peptides to immune cells, allowing for immune tolerance of healthy cells and the recognition and targeted killing of infected, tumorigenic and foreign cells [4]. The HLA genes are believed to be the most polymorphic in the human genome, with a total of 41,003 alleles currently recorded in the IPD‐IMGT/HLA Database (Release 3.59, January 2025) [5, 6]. Somewhat extraordinarily, the total estimated number of alleles in the worldwide population has been calculated to be around three million for each HLA class I gene [6].

The six classical HLA genes (*HLA‐A*, *‐B*, and *‐C* for class I and *HLA‐DQB1*, *‐DRB1*, and *‐DPB1* for class II) have been extensively studied for years and have been shown to play a vital role in the field of transplantation, disease association, and drug‐induced hypersensitivity. HLA class I and class II molecules are distinguishable from each other based on their genetic composition, protein structure, and function. There are, however, many HLA genes studied to a lesser extent, one group being the non‐classical HLA class I genes. These genes differ from classical HLA class I by their reduced genetic variability, heterogenous cellular expression patterns, and unique peptide presentation repertoires to different parts of the immune system.

In this review, we will provide a comprehensive overview of what is known about the structure and function of the non‐classical HLA class I molecule HLA‐E, in particular focusing on the genetics of *HLA‐E*, its allelic diversity, and provide a thorough and up‐to‐date overview of the published associations with haematopoietic cell transplantation (HCT) outcomes.

## 2 *HLA‐E* Structure and Function

The *HLA‐E* gene was identified in 1988 as the first non‐*HLA‐A*, *‐B*, or *‐C* class I gene [7, 8, 9]. The genetic structure of the *HLA‐E* gene is very similar to classical HLA class I genes, at approximately 3.5 kb in length, with eight exons. Exon 1 codes for the leader peptide, exons 2, 3, and 4 code for the α1, α2, and α3 domains of the protein respectively, exon 5 codes for the transmembrane region, and exons 6 and 7 code for the cytoplasmic tail [7, 8]. Together the α1 and α2 domains form the antigen recognition domain (ARD), which is responsible for creating appropriate hydrophobic and polar pockets to which specific residues within a peptide chain can interact and stabilise the molecule upon binding [10]. These exons encode the heavy chain of HLA‐E which, analogously to classical HLA class I heavy chain molecules, associates with a β_2_‐microglobulin light chain to form the complete HLA‐E molecule capable of presenting peptides on the cell surface [9].

The relative expression levels of HLA‐E molecules vary between cells and tissues; however, universally across all tissues, the transcriptional expression of *HLA‐E* is significantly reduced compared to classical HLA class I molecules [11, 12]. The other main disparity between HLA‐E and classical HLA class I molecules is its peptide binding repertoire. In most cases, HLA‐E is restricted to presenting peptides derived from the leader sequences of other HLA class I molecules [11, 13], whereas classical HLA molecules can present a widespread repertoire of peptides to enable immune recognition of almost any possible pathogenic antigen.

HLA‐E: peptide complexes also function within the immune system in a different manner to classical HLA class I molecules. HLA‐E: peptide complexes bind to the CD94/NKG2 family of C‐type lectin heterodimeric receptors found primarily on natural killer (NK) cells, but also on certain subsets of CD8^+^ T cells [14]. HLA‐E can bind to both the NKG2A and NKG2C members of this family, but with a six‐fold higher affinity for the CD94/NKG2A receptor [13, 14, 15, 16]. CD94/NKG2A contains immunoreceptor tyrosine‐based inhibitory motifs (ITIMs) in its cytoplasmic domain, which upon ligand binding, cause inhibition of NK cells' cytotoxic responses [17]. Conversely, CD94/NKG2C associates with the DAP‐12 adapter molecule containing immunoreceptor tyrosine‐based activating motifs (ITAMs), leading to NK cell activation [18]. The higher affinity of HLA‐E bound to HLA class I leader peptides for the inhibitory CD94/NKG2A receptor implies that in normal, homoeostatic conditions, HLA‐E binding induces inhibition of cytotoxic cellular responses. In doing so, it is acting as a signal to the immune system that the target cell is expressing classical HLA molecules and therefore is not tumorigenic or virally infected, sparing it from cell death.

## 3 Genetic Variation at the HLA‐E Loci

Early published research on HLA‐E focused on its basic functions in terms of: HLA class I leader peptide binding [11, 13, 16, 19, 20], interaction with NK cell receptors [14, 15, 17, 21] and genetic variation [22, 23, 24, 25, 26, 27, 28]. The majority of these genetic variation studies used sequencing strategies that only covered partial coding sequences (CDS), often just exon 2 and 3, the ARD of the molecule. As this region of the molecule has direct interactions with the peptide, it has often been thought that these exons contain polymorphisms that have the greatest impact on the function of HLA molecules. However, by sequencing only the CDS or selected exons, all genetic variation outside this region remained undetected.

Currently, *HLA‐E* has significantly less documented genetic diversity than the classical HLA class I genes, with 376 alleles described in the IPD‐IMGT/HLA Database (Release 3.59, January 2025) [5]. In comparison, there are 8556, 10,346, and 8657 alleles documented for *HLA‐A*, *‐B*, and *‐C* respectively [5]. Early *HLA‐E* studies looked at genetic variation in individual exons up to exons 2–3 as they encode the extracellular domains of the molecule including the ARD. Initial characterisation of classical HLA class I genes similarly only included these exons and yet significant genetic variation was observed between individuals with over 1500 classical HLA class I alleles reported in the IPD‐IMGT/HLA Database in 2005 (Release 2.8). In comparison, by 2005, only five *HLA‐E* alleles had been identified, corresponding to what are now known to be HLA‐E*01:01, HLA‐E*01:03:01, HLA‐E*01:03:02, HLA‐E*01:03:03 and HLA‐E*01:04 [7, 26, 29, 30].

Across all studies of *HLA‐E* genetic variation since its identification, two protein variants have been observed with consistent and high frequencies: HLA‐E*01:01 and HLA‐E*01:03. Together these alleles account for between 94% and 100% of alleles observed in cohorts; this combined frequency appears consistent across a significant number of ethnicities studied so far [31, 32, 33, 34, 35, 36, 37]. These two alleles differ by the single nucleotide polymorphism (SNP) A>G in exon 3, genomic DNA position (g.) 756 (counting from the first base of exon 1). This causes a non‐synonymous substitution in codon 107, changing AGG (Arginine) in HLA‐E*01:01 to GGG (Glycine) in HLA‐E*01:03 [8, 26]. An amino acid change from Arginine, bulky and positively charged, to Glycine, small and non‐polar, could be expected to induce significant changes in the protein structure. However in HLA‐E, this amino acid substitution only causes minor changes in the local structure around residue 107 and has very little impact on the structure of the peptide binding groove and overall molecule [38]. Despite these subtle changes in structure, the HLA‐E*01:03:peptide molecule has increased thermal stability causing a significant increase in cell surface expression compared to HLA‐E*01:01 molecules [38, 39].

Throughout the HLA community, the initial belief was that only non‐synonymous polymorphisms in the CDS resulted in functional differences, although both synonymous and intronic variations are now known to have an impact on the expression and function of HLA [40, 41]. Consequently, many studies investigating the association of *HLA‐E* genotypes with disease prevalence or prognosis simplified *HLA‐E* analysis models to only consider the two most prevalent alleles, HLA‐E*01:01 and HLA‐E*01:03 [42, 43, 44, 45, 46, 47, 48, 49]. While the combined frequency of HLA‐E*01:01 and HLA‐E*01:03 is consistent and high between studies, the frequency of each allele differs more noticeably. HLA‐E*01:01 has been found at frequencies of 24%–72% and similarly, HLA‐E*01:03 has been reported at frequencies of 28%–76%. This variability is potentially due to the ethnicity of the study cohort, patient disease [50] and/or regions of the gene covered in the different typing strategies used. There are sufficient *HLA‐E* allele frequency and ethnicity data to be able to highlight trends in global *HLA‐E* variation discovered thus far. It has been well‐established for many years that ethnicity is a significant factor in the variation of classical HLA [51]. Considering the lack of high‐resolution genotyping and the near bi‐allelic nature of *HLA‐E*, however, this might not be as trivial to observe for *HLA‐E* until more higher‐resolution genotyping data is available.

## 4 Population Differences in HLA‐E Allele Frequencies

**FIGURE 1:**
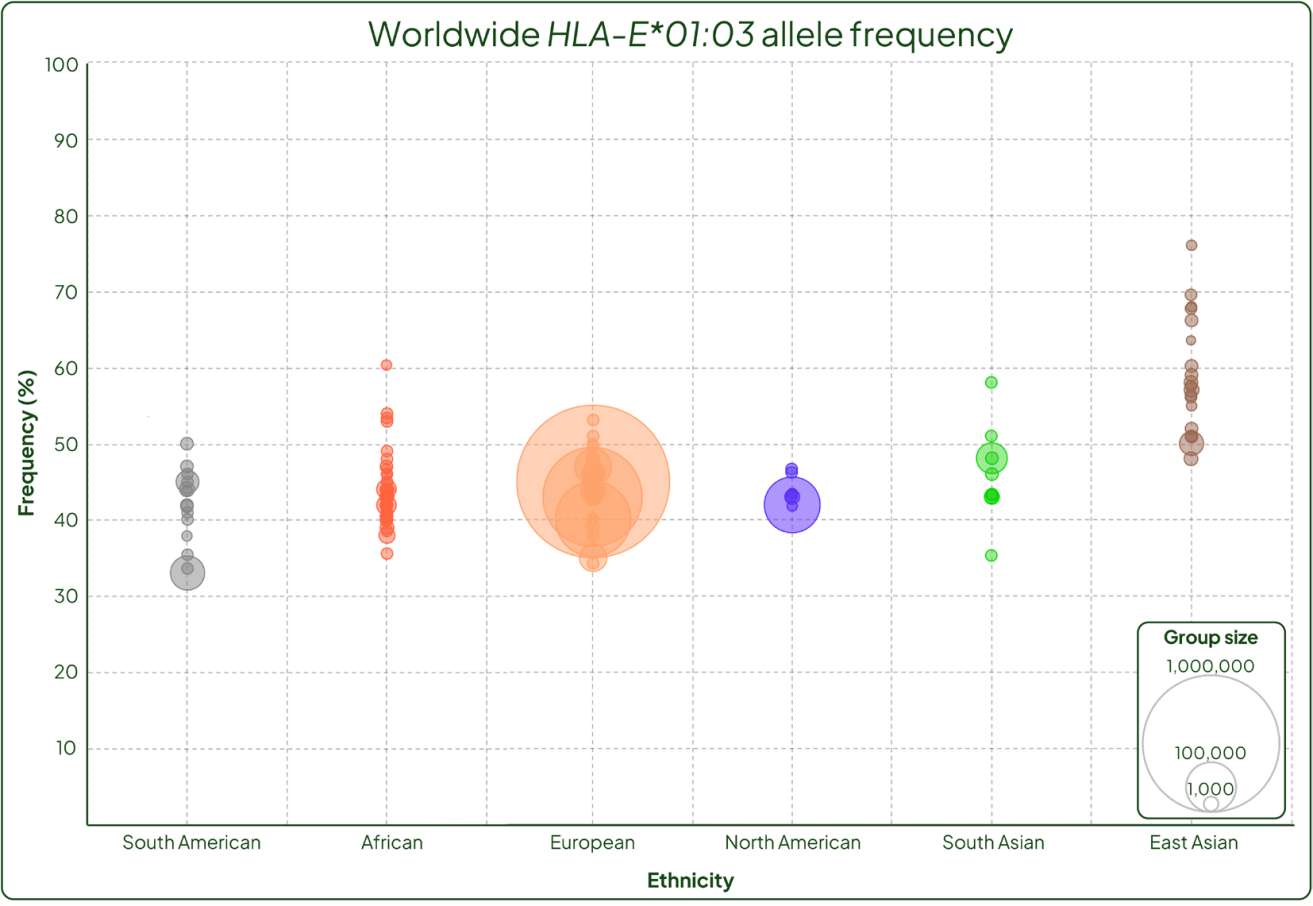
Worldwide HLA‐E*01:03 allele frequency grouped by ethnicity. South American cohorts are shown as grey bubbles, African as red, European as coral, North American as violet, South Asian as green and East Asian as brown. Bubble area represents the cohort size. (Made with Piktochart).

**FIGURE 2:**
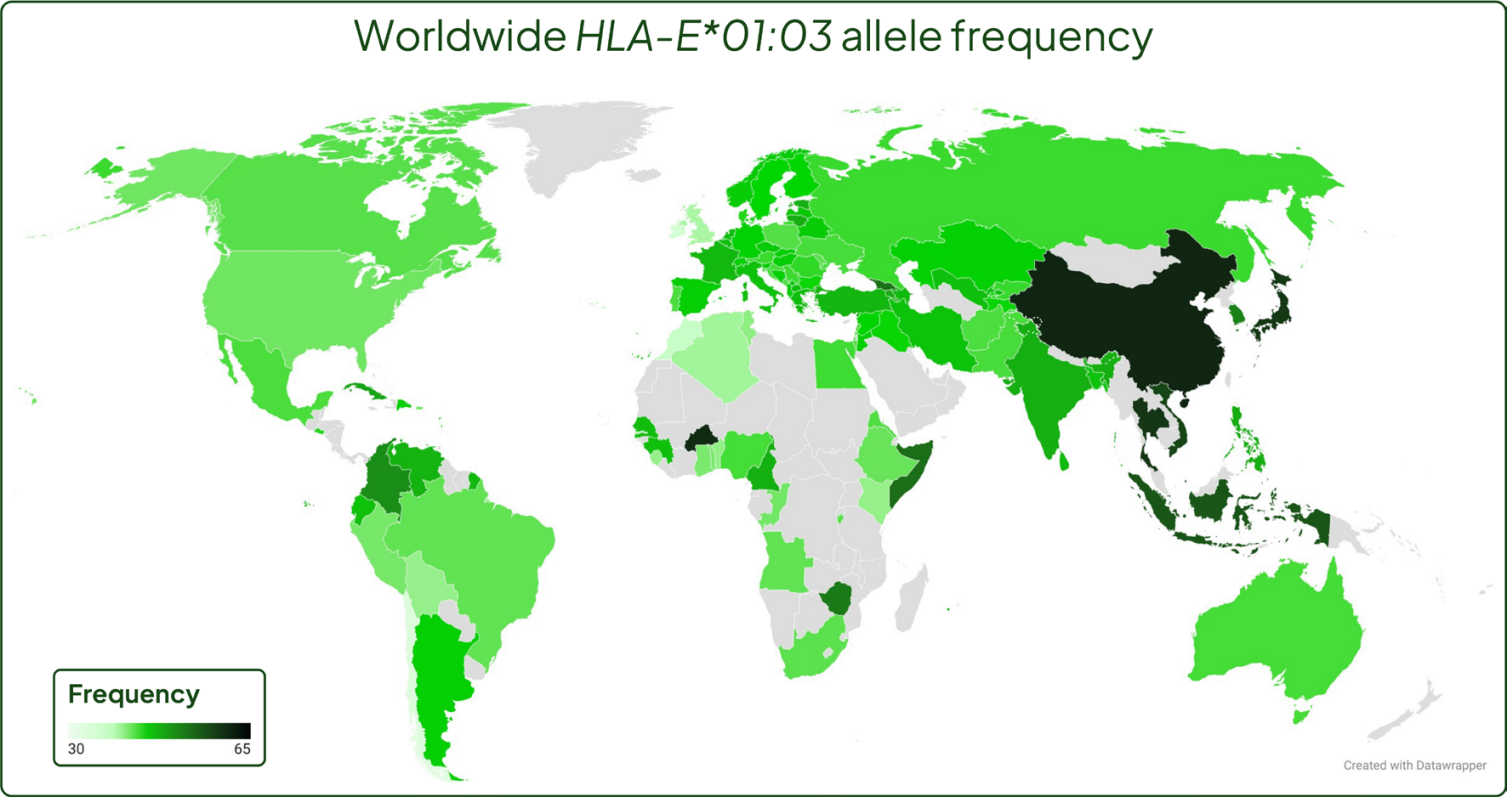
A world map overlayed with HLA‐E*01:03 allele frequency depicted by variation from light green to dark green; low to high allele frequency. Countries with no data are shaded grey. This displays the increasing frequency of HLA‐E*01:03 moving from Europe across to Asia and in particular East Asia. (Made with Datawrapper).

Based on the current available literature, HLA‐E*01:03 appears to be present at higher frequencies in East Asian populations ranging from 48% to 76% [24, 26, 28, 34, 35, 48, 52, 53, 54, 55] and lower in South American, North American and European populations, ranging from 34% to 53% [28, 29, 32, 35, 37, 56, 57, 58, 59, 60, 61, 62, 63, 64, 65] respectively (Figure 1). Calculating the mean weighted average based on sample sizes and plotting this for each country on a world map allows for visualisation of this pattern across the globe (Figure 2). Although many countries have no *HLA‐E* allele frequency data, a pattern is visible where Europe and the Americas generally have a lower HLA‐E*01:03 frequency, with the allele frequency increasing towards Asia and reaching a maximum in East Asia (Figures 1 and 2). Of the African cohorts of individuals typed for HLA‐E so far, the mean frequency of HLA‐E*01:03 across the continent appears to be close to 50% but demonstrates greater variation than other geographic regions ranging from Burkina Faso in West Africa (60%) to Tunisia in the north (36%) (Figures 1 and 2). This concurs with the generally accepted concept that Africa has greater genetic diversity than other regions; however, given that a large proportion of countries in Africa have no data, and the potential for these data to be skewed by lack of numbers, this data should be interpreted with caution [66]. A mirrored trend also exists for HLA‐E*01:01, observed at higher frequencies in American and European sample groups and lowest in East Asian groups. The caveat to this data is that sample sizes are small for many groups (60% of groups are smaller than 500 samples), so caution should be used before making inferences about whole populations.

**FIGURE 3:**
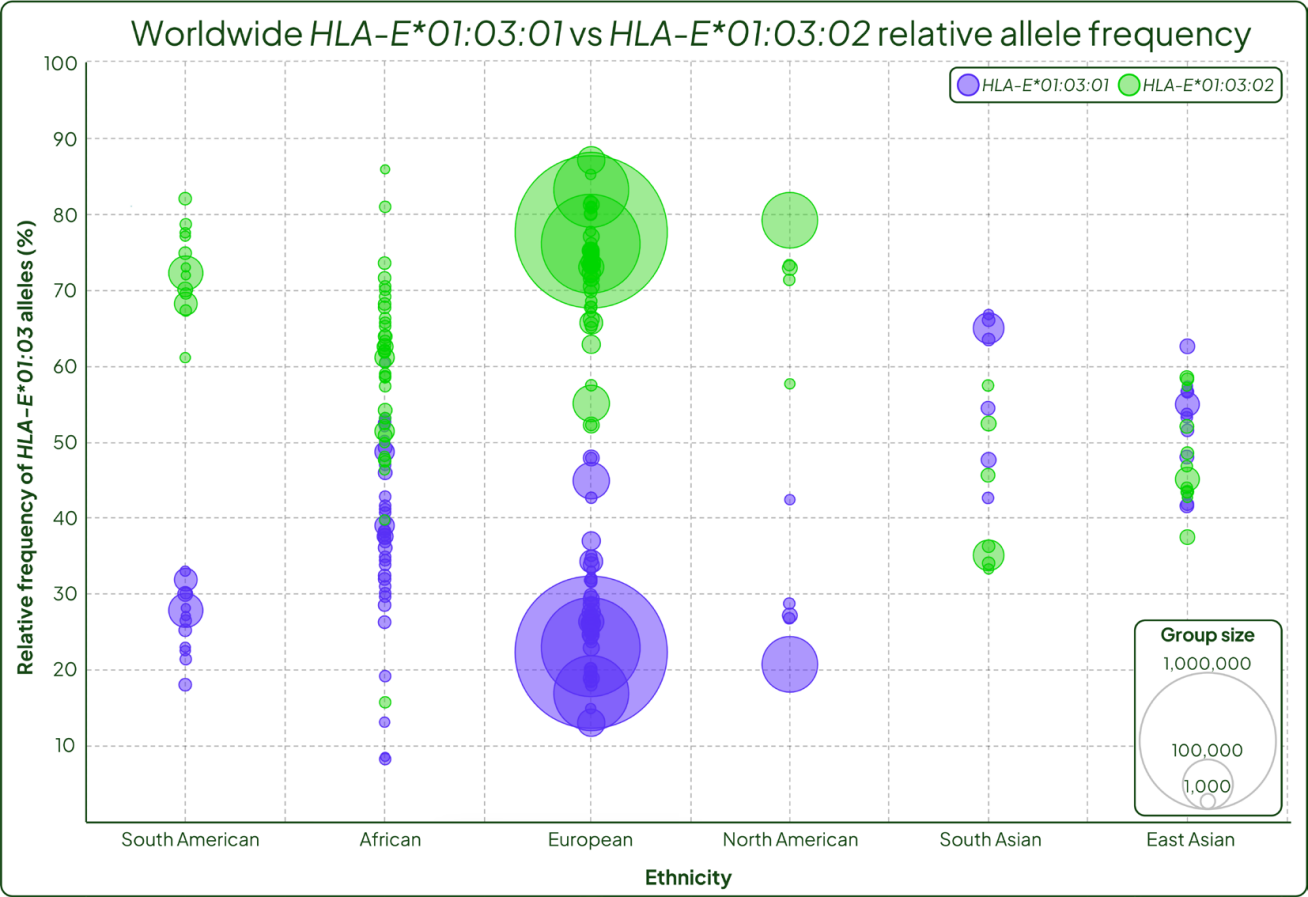
HLA‐E*01:03:01 and HLA‐E*01:03:02 relative allele frequency grouped by ethnicity. Calculated as the proportion of the total HLA‐E*01:03 frequency in each cohort. Violet bubbles represent HLA‐E*01:03:01 allele frequency, green bubbles represent HLA‐E*01:03:02 allele frequency. Bubble area represents the cohort size. (Made with Piktochart).

Within the HLA‐E*01:03 allele group, there are two highly prevalent synonymous alleles, HLA‐E*01:03:01 and HLA*01:03:02 which differ in exon 2 at g.424C>T. Interestingly, this is not mirrored in the HLA‐E*01:01 allele group, where the corresponding synonymous allele with this SNP (HLA‐E*01:01:02) has been observed at a maximum frequency of 0.3% [35]. Comparing the proportion of HLA‐E*01:03 alleles that are either HLA‐E*01:03:01 or HLA‐E*01:03:02 also reveals geographic patterns of frequency differences. The relative frequency of HLA‐E*01:03:01 versus HLA‐E*01:03:02 clearly differentiates in South American, European, and North American sample groups, where HLA‐E*01:03:02 is consistently more frequently observed than HLA‐E*01:03:01 in these ethnicities (ranging from 52% to 87% compared to 13%–48% respectively) (Figure 3). A similar observation can also be made in African populations, although there is more variability and overlap across sample groups (HLA‐E*01:03:02 16%–86% and HLA‐E*01:03:01 8%–60%; Figure 3). Asian populations demonstrate opposite patterns of relative frequencies, with HLA‐E*01:03:01 being observed more frequently. This is more evident in South Asian ethnicities (ranging from 43% to 67%) than East Asian, where there is greater overlap of the observed frequency of the two alleles (Figure 3).

HLA‐E*01:04 was first reported in 1990 and identified as differing from HLA‐E*01:03 in exon 3 at g.906A>G, R157G [26]. It was found once in a small cohort of 11 Japanese unrelated blood donors that were investigated to determine if HLA‐E variation existed within this population [26]. Several studies have actively tried to detect this allele without success, and on occasion potential sequence‐specific oligonucleotide (SSO) or sequence‐specific primer (SSP) based detection of HLA‐E*01:04 was checked by SBT and shown to be erroneous [23, 56, 64, 67]. It has been proposed that HLA‐E*01:04 might be a sequencing artefact and not a real allele; however, without re‐sequencing the original sample, this cannot be confirmed [68].

## 5 Non‐Coding Variation Identified by Full‐Gene HLA‐E Sequencing

More recently, reports have been published that utilise full‐gene sequencing of *HLA‐E*; however, there are only a few such studies, and typically cohort sizes are small [57, 63, 69, 70, 71]. One recent paper describes *HLA‐E* variation in a very large cohort of over 2.5 million volunteer haematopoietic cell donors [35]. From this work, 345 novel *HLA‐E* alleles were identified, and 170 of those were submitted to the IPD‐IMGT/HLA Database, which resulted in a significant increase in the documented variation within *HLA‐E* [72]. Given the very large cohort size, however, this number of new variants does not appear to be suggestive of a similar extent of genetic variation that has been observed in the classical HLA class I loci. A possible reason for this could be the sequencing strategy used in this study for their routine HLA‐E typing, which only included parts of exon 2 to exon 3, covering a total of 535 base pairs including the region that distinguishes the HLA‐E*01:01 and HLA‐E*01:03 alleles [72]. More recently, another large study describing the variation of *HLA‐E* in over 6000 samples was published, which utilised full‐gene sequencing [73]. Compared to the study by Paech et al. [72], the identification of novel genetic variation reported in this study was over 30 times higher, with a total of 86 novel alleles, suggesting there is more variation in the additional exonic and non‐coding regions of the gene identified by using the full gene sequencing strategy than had been previously anticipated [73]. Additionally, there may be differences in the ethnicity of individuals included in each study that have affected the amount of variation observed, with both under‐represented populations and less typing of HLA‐E overall both affecting the data quality. We hypothesise that with continued full‐gene sequencing in larger and more ethnically diverse cohorts, the genetic diversity of *HLA‐E* will be greater than initially suggested.

**FIGURE 4:**
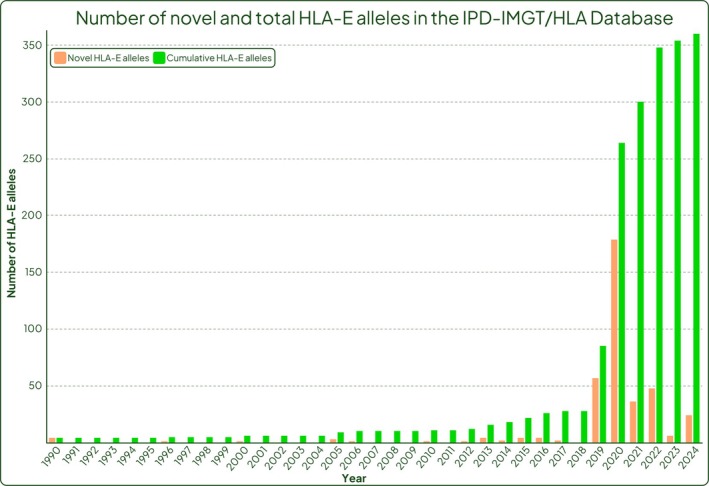
Cumulative number of *HLA‐E* alleles in the IPD‐IMGT/HLA Database up to 2025 (Release 3.59). Coral bars represent the number of novel *HLA‐E* alleles released per year, green bars represent the cumulative number of *HLA‐E* alleles.

As there appears to be a stabilising selection on HLA‐E, making HLA‐E*01:01 and HLA‐E*01:03 account for the majority of protein variants observed, it is possible that HLA‐E genetic variation has arisen more frequently in non‐coding regions. A limitation of previous typing strategies was that any variation in non‐coding regions would likely have been missed. In 2006 Pyo et al. [74] published the first full‐gene *HLA‐E* sequencing data on a cohort of 33 cell lines, in doing so finding the first two intronic *HLA‐E* variants, HLA‐E*01:01:01:02 and HLA‐E*01:03:01:02 [74]. Yet it was not until recently that the number of studies using full‐gene *HLA‐E* sequencing has started to increase and this is now being reflected in the IPD‐IMGT/HLA Database where the last 5 years have seen a significant increase in the number of *HLA‐E* alleles submitted (Figure 4). As of 2025, there are a total of 141 intronic variants of *HLA‐E* often differing from their closest allele only by a SNP, highlighting the importance of using full‐gene sequencing in uncovering the full extent of genetic diversity.

The first two large batches of novel *HLA‐E* alleles released by the IPD‐IMGT/HLA Database occurred in 2019 with release 3.39 and 2020 with release 3.40 (Figure 4). The differences between these two batches highlight how large datasets can skew the apparent genetic variability within a gene. In release 3.39, 39 newly identified novel *HLA‐E* alleles were described, 82% of which were non‐coding variants and the remaining 18% differed in exons, all of which were identified in our laboratory using the full‐gene *HLA‐E* sequencing method described by Lucas et al. [73]. In the subsequent release 3.40 of the IPD‐IMGT/HLA Database, 107 novel *HLA‐E* alleles were published and in contrast to release 3.39, only 7% of the newly described *HLA‐E* alleles were intronic variants, meaning the remaining 93% differed in exons. Again, all of these novel sequences were submitted by one submitter, DKMS Life Science Lab [72]. The *HLA‐E* genotyping strategy described by this group for bulk genotyping uses a short amplicon NGS method that sequences part of exons 2 and 3 [35].

**FIGURE 5:**
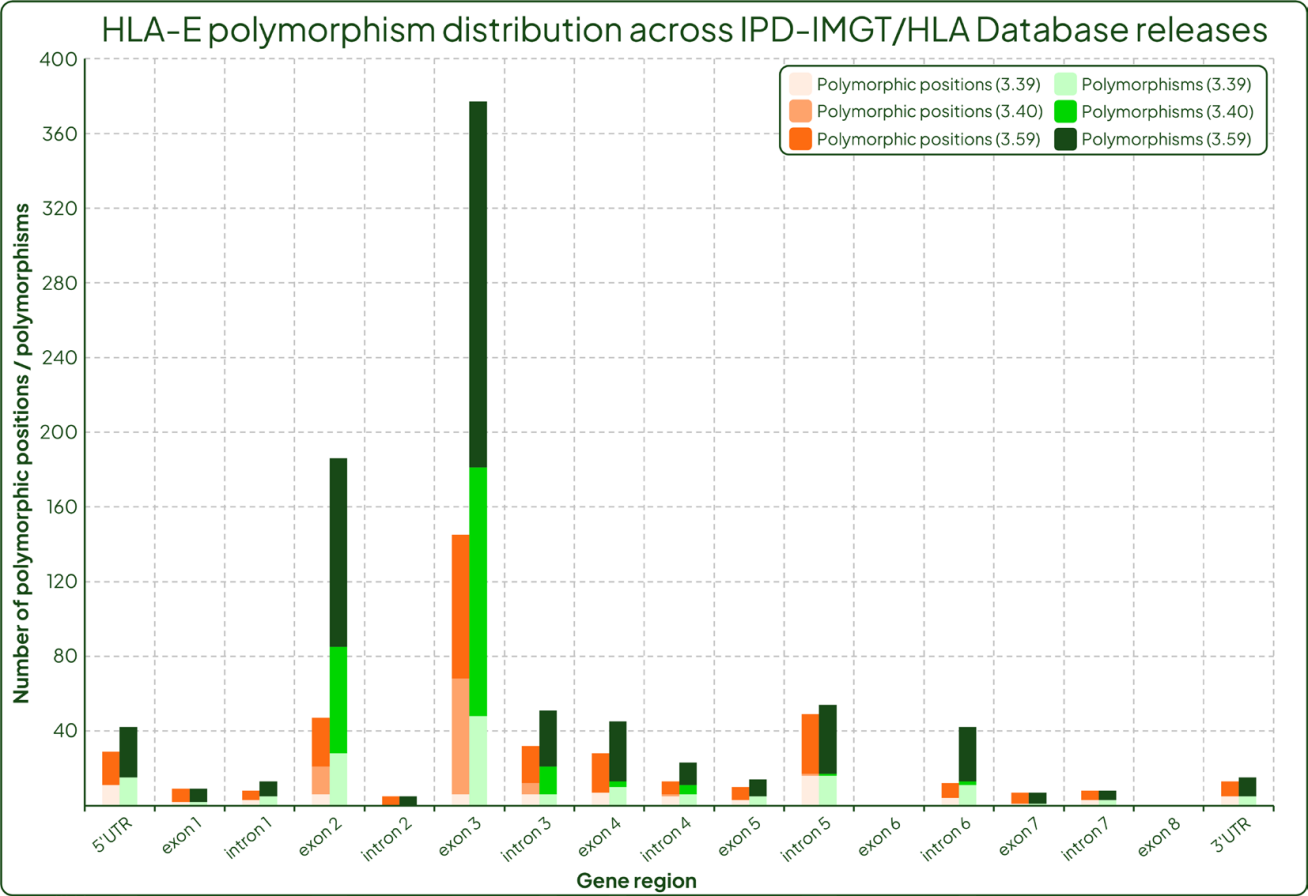
Genetic variation in each region of the *HLA‐E* gene. Coral bars represent the number of positions in each gene region that are polymorphic and green bars represent the total number of polymorphisms in that gene region. Lightest shades of each bar represent data from release 3.39 of the IPD‐IMGT/HLA Database, medium shades from release 3.40 and dark shades from release 3.59.

**FIGURE 6:**
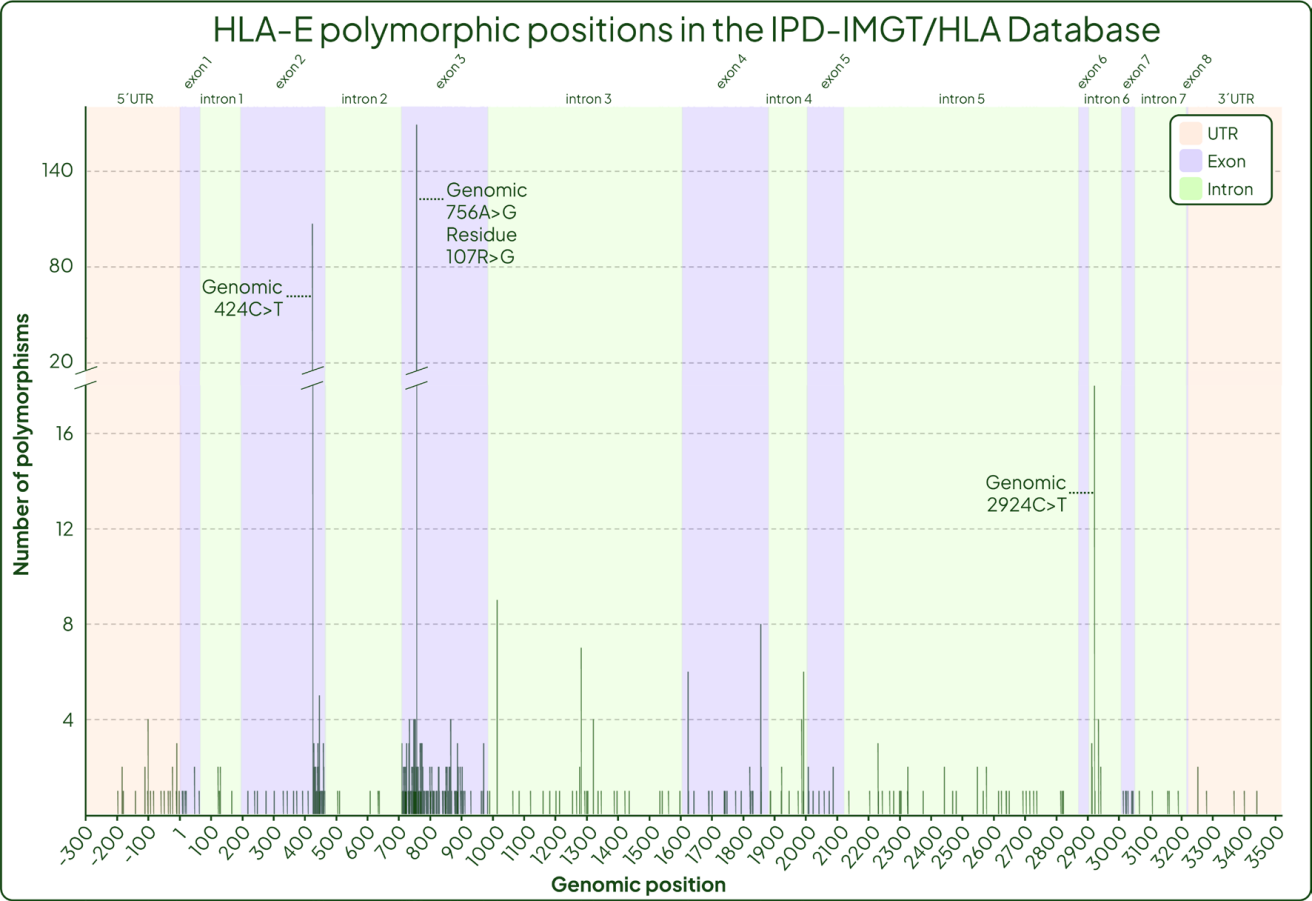
Number of polymorphisms at each genomic position throughout the *HLA‐E* gene. 5´UTR and 3´UTR regions are coral, exons violet and introns green. The number of polymorphisms is shown by dark green lines, data taken from release 3.59 of the IPD‐IMGT/HLA Database. The three highest frequency polymorphisms are labelled with their genomic position and amino acid changes if applicable.

Figure 5 shows the distribution of polymorphisms condensed into exons and introns taken from these two releases of the IPD‐IMGT/HLA Database, as well as the more recent release 3.59. There were 53 polymorphic positions in non‐coding regions and 25 in exons in release 3.39 compared to release 3.40 with 62 in non‐coding regions and 114 in exons. This enrichment of documented polymorphic positions in exons 2 and 3 between these two releases is explained by the short, targeted genotyping method used by this group (Figure 5). This is further visualised by Figure 6 which displays the number and position of *HLA‐E* polymorphisms in release 3.59 of the IPD‐IMGT/HLA Database. Of note are the high frequency SNPs 424C>T and 756A>G, and exons 2 and 3 containing the greatest total number of polymorphisms (counting every occurrence a position varies from the reference HLA‐E*01:01:01:01) due to the functional relevance that is likely to be correlated with these differences; but also, this is the region covered by the typing strategy utilised by the DKMS Life Science Lab.

Such typing strategies that target a short region are particularly advantageous for very high‐throughput processes and could be sufficient to select donors based on *HLA‐E* in the future, if one SNP is shown to be important in transplant outcomes, for example. The trade‐off is that such strategies cannot shed light on the full genomic variation of a gene and could lead to a skewed view of genetic diversity, as highlighted here for *HLA‐E*. Full‐gene sequencing has the advantage of capturing all variation within a gene, improving our knowledge of its true genetic diversity and facilitating analyses into genomic variation with the caveat of being more resource intensive. Overall, care should be taken when reviewing *HLA‐E* variation given the relatively small number of documented *HLA‐E* alleles and the potential for it to be skewed by large datasets. Understanding the genetic composition and allele frequencies of *HLA‐E* is important if we want to study its impact in complex immune environments such as transplantation.

## 6 *HLA‐E* in Haematopoietic Cell Transplants

Selecting the most appropriate unrelated donors for patients undergoing HCT requires characterisation of several key factors including their *HLA* genes, cytomegalovirus (CMV) serostatus, age, sex at birth and ABO type, as well as other non‐clinical considerations such as donor availability. Historically, patients and their unrelated donors were HLA matched for five classical *HLA* genes: *HLA‐A*, *‐B*, *‐C*, *‐DRB1* and ‐*DQB1*, known as a 10/10 HLA match [75, 76, 77]. This was generally accepted and practised as the'bes' *HLA* match for many years. In the last decade, however, benefits of matching for a sixth gene, *HLA‐DPB1*, have been shown in relation to patient outcomes [78, 79]. Consequently, a 12/12 HLA match including *HLA‐DPB1* is now considered the gold standard for HCT unrelated donor selection [80]. The probability of finding a 12/12 HLA matched donor is often lower than a 10/10 HLA match due to the presence of a recombination hotspot located between *HLA‐DQB1* and *HLA‐DPB1* [81]. For patients with less common *HLA* types, it can be challenging to identify either a 12/12 or a 10/10 HLA match; in these cases, other selection factors have been shown to possibly compensate for the *HLA* mismatches, including CMV matching, permissive *HLA‐DPB1* mismatching, or some studies have even suggested selecting for certain non‐*HLA* genes [77, 78, 79, 82, 83]. To date, non‐classical HLA class I genes have not routinely been included in HCT donor selection strategies, although it has been suggested *HLA‐E* could play a role in the successful outcomes of HCT and solid organ transplantation given its function in both innate and adaptive immune responses [60, 84].

There are limited numbers of studies looking into the impact of *HLA‐E* matching in HCT outcomes, and currently there is no clear consensus as to if, or how, *HLA‐E* should be included in donor selection strategies. In 2005, the first study investigating the impact of *HLA‐E* on HCT outcome was published [84]. Since then, a total of 19 studies have been published covering an assortment of donor types, ethnicities, and conditioning regimens; the findings of which are summarised in Table 1. All studies to date have been retrospective analyses. Despite the current era of widespread next‐generation sequencing (NGS) HLA typing methods, little has been written about the in‐depth nature of *HLA‐E* genetics in the context of HCT, often simplifying allelic diversity to only two protein variants, which appears not to be a true representation of the genetic diversity.

**TABLE 1:** Summary of studies into *HLA‐E* genotype impact on HCT outcomes.

Publication	HLA‐E genotyping method	Cohort—HLA matching	Diagnosis	Conditioning	T‐cell depletion	*HLA‐E* matched	*HLA‐E* factor	Infection	GvHD	NRM	Relapse	DFS	Overall survival
Tamouza et al. [84]	Exon 3 RFLP, SSP	77 MUD—10/10	26% CL, 35% AL, 27% BM failure, 12% other	100% MAC	22% deplete, 78% replete	61.0%	Donor HLA‐E*01:03	↓ Bacterial HLA‐E*01:03, 01:03, HR 1.0, HLA‐E*01:01, 01:01, HR 2.20; 95% CI 1.90–2.56; *p = 0.03*	N/A	↓ HLA‐E*01:03, 01:03, HR 1.0, HLA‐E*01:01, 01:01, HR 2.12, 95% CI 1.01–4.46; *p = 0.048*	N/A	N/A	N/A
Tamouza et al. [47]	Exon 3 SBT	187 Identical sibling donors	26% CL, 59% AL, 15% other	100% MAC	100% replete	100%	Patient or donor HLA‐E*01:03, *01:03*	N/A	↓ Acute II–IV HLA‐E*01:03, 01:03, HR 1.0, HLA‐E*01:01, 01:03 or HLA‐E*01:01, 01:01, HR 1.4, 95% CI 1.1–1.8, *p = 0.009*	↓ HLA‐E*01:03, 01:03, HR 0.42, 95% CI 0.17–0.91, *p = 0.04*	N/A	N/A	↑ HLA‐E*01:03, 01:03, HR 0.52, 95% CI 0.25–1.11, *p = 0.09*
Danzer et al. [42]	Exon 3 RT‐PCR	83 MUD—10/10	7.2% CML, 66.7% AML, 13.1% MDS, 13.1% other	56.6% MAC, 43.4% RIC	100% replete	89.2%	Patient HLA‐E*01:03, *01:03*	N/A	↓ Acute III–IV HLA‐E*01:01, 01:01 or HLA‐E*01:01, 01:03 versus HLA‐E*01:03, 01:03, 39% versus 17%, *p* = 0.09*	↓ HLA‐E*01:03, 01:03 versus HLA‐E*01:01, 01:01, 6% versus 41%, p = 0.01*	N/A	↑ HLA‐E*01:03, 01:03, HR 3.09, 95% CI 1.57–6.09, *p = 0.001*	↑ HLA‐E*01:03, 01:03, *HR 1.12, 95% CI 0.31–1.94, p* = 0.006
Ludajic et al. [45]	Exon 3 SBT	124 MUD—10/10 + DRB3/4/5	28.2% CML, 29.8% AML, 15.3% ALL, 11.3% MDS, 15.3% other	73% MAC, 27% RIC	24% deplete, 76% replete	53.0%	Donor HLA‐E*01:03, 01:03	N/A	↓ Acute II–IV HR 0.39, 95% CI 0.16–0.99, *p* = 0.047	↑ HR 3.94, 95% CI 1.03–15.02 *p* = 0.045	↑ HR 2.24, 95% CI 1.03–4.88, *p* = 0.042	N/A	N/A
							Donor HLA‐E*01:01, 01:03	N/A	↓ Chronic HR 0.36, 95% CI 0.14–0.90, *p* = 0.030	N/A	N/A	N/A	N/A
Bogunia‐Kubik et al. [85]	Exon 3 unknown	100 MUD—10/10, identical sibling donors, haploidentical	N/A	N/A	N/A	N/A	Patient HLA‐E*01:03, 01:03	N/A	N/A	N/A	N/A	N/A	↑ 100% versus 61%, *p* = 0.045*
							*HLA‐E* mismatch	N/A	↑ Acute II–IV 5 8% versus 35%, *p* = 0.047*	N/A	N/A	N/A	N/A
Bogunia‐Kubik et al. [86]	Exon 3 unknown	55 MUD—10/10, 45 identical sibling donors	87% haematological malignancies, 10% inborn errors, 3% anaemia	41% MAC, 59% RIC	N/A	N/A	Patient HLA‐E*01:03, 01:03	N/A	N/A	N/A	N/A	N/A	↑ 100% versus 55%, *p* = 0.095*
							*HLA‐E* mismatch	↓ Herpes virus *0%* vs *24%, p* = 0.035*	↑ Acute II–IV 67% versus 34%, *p* = 0.040*	N/A	N/A	N/A	N/A
Fürst et al. [87]	Exon 2 + 3 SSP	116 MUD—10/10	27.6% CML, 31.9% AML, 19.8% ALL, 6.9% MDS, 13.8% other	77.6% MAC, 22.4% RIC	0.9% deplete, 99.1% replete	68.1%	N/A	N/A	N/A	N/A	N/A	N/A	N/A
Harkensee et al. [88]	SNP analysis	460 mMUD—various	100% AL	100% MAC	100% replete	N/A	*HLA‐E* mismatch	N/A	N/A	N/A	N/A	N/A	↓ *p* = 0.023*
Hosseini et al. [43]	Exon 3 SSP	12 MUD—10/10, 33 identical sibling donors, 11 HLA‐ haploidentical killer Ig‐like receptor mismatched	59% AML, 23% ALL, 18% other	39.3% MAC, 60.7% RIC	19.6% deplete, 80.4% replete	100%	Patient or donor HLA‐E*01:03, 01:03	N/A	↓ Acute II–IV HLA‐E*01:03, 01:03, HR 1.0, HLA‐E*01:01, 01:01 or HLA‐E*01:01, 01:03, HR 1.2, 95% CI 1.03–1.5, *p* = 0.02 Chronic *p* = 0.04	N/A	N/A	N/A	↑ HLA‐E*01:03, 01:03, HR 0.15, 95% CI 0.04–0.51, *p* = 0.0023
Hosseini et al. [89]	Exon 3 SSP	45 MUD—12/12, 11 haploidentical	59% AML, 23% ALL, 18% other	39.3% MAC, 60.7% RIC	19.6% deplete, 80.4% replete	100%	Patient or donor HLA‐E*01:03, 01:03	N/A	N/A	N/A	↓ HLA‐E*01:03, 01:03, HR 0.1, 95% CI 0.01–0.95, *p* = 0.004	↑ HLA‐E*01:03, 01:03, HR 0.15, 95% CI 0.04–0.51, *p* = 0.002	N/A
Zhu et al. [49]	Exon 3 SBT	119 Identical sibling donors	N/A	N/A	N/A	100%	Patient or donor HLA‐E*01:03	↓ CMV 37.9% versus 62.5% *p* = 0.0295*	N/A	N/A	N/A	N/A	N/A
Mossallam et al. [90]	Exon 3 RFLP‐PCR	88 Identical sibling donors	23.9% CML, 59.1% AML, 17% MDS	81.8% MAC, 18.2% RIC	100% replete	100%	Patient or donor HLA‐E*01:03, 01:01 or HLA‐E*01:03, 01:03	N/A	N/A	N/A	↓ HR 0.30, 95% CI 0.91–1.69, *p* = 0.09	N/A	N/A
Tsamadou et al. [91]	Exon 2 + 3 SBT	509 MUD – 10/10	12.6% AL, 61.5% AML, 25.9% ALL	67.8% MAC, 32.2% RIC	63.2% deplete, 36.8% replete	62.9%	Patient HLA‐E*01:03, 01:03	N/A	N/A	↑ HR 1.74, 95% CI 1.09–2.78, *p* = 0.02	N/A	↓ HR 1.47, 95% CI 1.04–2.07, *p* = 0.03	↓ HR 1.45, 95% CI 1.00–2.10, *p* = 0.05
							*HLA‐E* mismatch	↓ 9.5% versus 17.2%*	↓ Acute II‐IV 7.7% versus 12.3%* Chronic HR 0.7, 95% CI 0.47–1.04, *p = 0.074*	↓ HR 0.63, 95% CI 0.43–0.91, *p* = 0.015	N/A	↑ HR 0.71, 95% CI 0.55–0.92, *p* = 0.008	↑ HR 0.63, 95% CI 0.43–0.83, *p* = 0.001
Tsamadou et al. [92]	Exon 2 + 3 NGS	1840 MUD—10/10	75% AML, 25% ALL	76.9% MAC, 23.1% RIC	29.4% deplete, 70.5% replete	67.5%	Donor HLA‐E*01:03, 01:03	N/A	N/A	N/A	↑ HR 1.35, 95% CI 1.08–1.69, *p* = 0.0083	↓ HR 1.28, 95% CI 1.09–1.51, *p* = 0.0027	N/A
							Donor HLA‐E*01:03, 01:03 T‐cell replete subset	N/A	N/A	↑ HR 1.41, 95% CI 1.11–1.81, *p* = 0.0058	N/A	↓ HR 1.35, 95% CI 1.14–1.60, *p* = 0.0006	N/A
Kordelas et al. [44]	Exon 3 SSP	66 MUD—unknown, 27 related	59.1% AML, 5.4% ALL, 10.8% MDS, 24.7% other	100% MAC	58.1% deplete, 41.9% replete	N/A	Donor HLA‐E*01:03, 01:03	N/A	N/A	N/A	N/A	N/A	↓ HR 3.0, 95% CI 1.1–7.8, p = 0.0237*
Mardani et al. [46]	Exon 3 SSP	200 Identical sibling donors	51% AML, 30% ALL, 19% other	100% MAC	100% deplete	N/A	Patient or donor HLA‐E*01:03	N/A	N/A	N/A	↑ HR 2.50, 95% CI 0.32–19.20, *p* = 0.37	N/A	N/A
Siemaszko et al. [93]	SNP analysis	78 MUD, 19 sibling, 3 haploidentical	38% ALL, 9% ALL, 9% SAA, 6% MDS, 5% WAS, 4% CML, 4% JMML, 25% other	18% MAC, 69% RIC, 13% NMA	N/A	73%	*HLA‐E* mismatch	N/A	↑ Acute I–IV 71.4% versus 56.6%*	N/A	N/A	N/A	N/A
							Patient HLA‐E*01:01, 01:01	↑ CMV 67.7% versus 53.0%, *p* = 0.050*	N/A	N/A	N/A	N/A	N/A
Petersdorf et al. [94]	SNP analysis	1,629 haploidentical	39% AML, 17% ALL, 17% MDS/MPN, 11% lymphoma, 4% CML, 2% myeloma, 4% other	43% MAC, 49% RIC/NMA, 9% unknown	96% deplete, 3% replete	N/A	Patient HLA‐E*01:03, 01:03	N/A	N/A	↓ HR 0.58, 95% CI 0.40–0.85, *p = 0.005*	N/A	N/A	↑ HR 0.70, 95% CI 0.56–0.88, *p = 0.002*
Petersdorf et al. [95]	SNP analysis	3,706 single HLA mMUD	N/A	N/A	N/A	55%	Patient HLA‐E*01:03, 01:03	N/A	N/A	N/A	↑ HR 1.20, 95% CI 1.01–1.43, *p = 0.04*	↓ HR 1.18, 95% CI 1.07–1.30, *p < 0.001*	N/A
Petersdorf et al. [96]	SNP analysis	903 single HLA‐B mMUD	N/A	N/A	N/A	53%	Donor HLA‐E*01:01, 01:03	N/A	↑ Acute II–IV HR 1.49, 95% CI 1.01–2.20, *p = 0.04*	N/A	N/A	N/A	N/A
							Donor HLA‐E*01:03, 01:03	N/A	N/A	N/A	N/A	↓ HR 1.30, 95% CI 1.02–1.66, *p = 0.03*	N/A
							HvG mismatch	N/A	N/A	N/A	↓ HR 0.63, 95% CI 0.41–0.98, *p = 0.04*	N/A	N/A

*Note:* Up and down arrows indicate an increase and decrease in the outcome respectively. Green or red shading represents desirable or unfavourable changes to outcomes respectively, light shading indicates where only a trend (*p* > 0.05), not significance (*p* ≤ 0.05) was observed. All *HLA‐E* matching was done to 2nd field resolution by matching across the gene regions listed for study. All reported *p* values are from multivariate analyses apart from *which indicates univariate analysis.

Abbreviations: AL, acute leukaemia; ALL, acute lymphoblastic leukaemia; AML, acute myeloid leukaemia; BM, bone marrow; CI, confidence interval; CL, chronic leukaemia; CML, chronic myeloid leukaemia; HR, hazard ratio; HvG, host‐versus‐graft; MAC, myeloablative conditioning; MDS, myelodysplastic syndrome; mMUD, mismatched unrelated donor; MUD, matched unrelated donor; NMA, non‐myeloablative; RIC, reduced‐intensity conditioning.

### 6.1 Protective Role of HLA‐E*01:03

The first published study on the potential impact of HLA‐E genotypes in HCT outcome was in a cohort of 77 patients and their 10/10 HLA matched unrelated donors (MUD), where 78% of patients received a T‐cell replete transplant and 61% of pairs were found to be *HLA‐E* matched [84]. The authors concluded that patients receiving a graft from an HLA‐E*01:03 positive donor significantly reduced the risk of non‐relapse mortality (NRM) [84]. It was also found that the presence of HLA‐E*01:03 in the donor's genotype (HLA‐E*01:01, 01:03, or HLA‐E*01:03, 01:03) reduced the risk of severe bacterial infection post‐transplant compared to HLA‐E*01:01, 01:01 donors [84]. A small study on 119 sibling transplant pairs suggested that HLA‐E*01:03 was associated with a reduced rate of CMV infection compared to HLA‐E*01:01, 01:01 genotypes [49]. Similarly, a more recent study on 100 paediatric HCT patients and their allogeneic donors (78 MUD, 19 sibling, 3 haploidentical) also found the absence of HLA‐E*01:03 in the patient was associated with an increase in CMV infection (HLA‐E*01:01, 01:01 68% vs. HLA‐E*01:01, 01:03 or HLA‐E*01:03, 01:03 53%) [93].

Not all studies have been able to confirm the beneficial impact of HLA‐E*01:03 on viral infection risk. A subsequent study in 187 HLA identical siblings all receiving T‐cell replete transplants failed to verify these findings [47]. This later study also reported that patients and correspondingly their HLA identical sibling donors that were homozygous for the HLA‐E*01:03 allele incurred significantly decreased rates of grade II‐IV acute graft‐versus‐host disease compared to any other genotype combination and also reported possible improved overall survival (OS) probabilities [47].

In a smaller study of 83 patients receiving a T‐cell replete, 10/10 HLA, MUD transplant, Danzer et al. [42] corroborated the protective associations of patient HLA‐E*01:03, 01:03 genotypes on aGvHD, NRM and OS probabilities but also suggested that the genotype was associated with increased disease‐free survival (DFS) risk [42]. The authors did not include donor *HLA‐E* genotype in their analysis because 89% of pairs in this study were *HLA‐E* matched; therefore, it was assumed, but not statistically proven, that the same observations would be found when analysing the donors' genotypes. Analogously, similar results were seen in another two small studies of 56 related and 10/10 HLA MUD transplants, where all patients were *HLA‐E* matched and 80% received a T‐cell replete transplant [43, 89]. Here, the patient and donor HLA‐E*01:03, 01:03 genotype was also significantly associated with decreased risk of aGvHD, chronic GvHD (cGvHD), and disease relapse, while both DFS and overall survival were improved [43, 89]. The protective effect of patient HLA‐E*01:03, 01:03 genotype was also reported in two published abstracts from one group. It was reported that in a cohort of 100 10/10 HLA, related and MUD transplants, improved overall survival was observed in patients with the HLA‐E*01:03, 01:03 genotype, although it is not clear if these two abstracts are from independent cohorts [85, 86].

The effect of the HLA‐E*01:03, 01:03 genotype was both beneficial and detrimental in the study by Ludajic et al. [45]. In this cohort of 124 10/10 and DRB3/4/5 HLA MUD transplants, where 76% received T‐cell replete transplants and 53% of pairs were *HLA‐E* matched, donor HLA‐E*01:03, 01:03 genotype was similarly associated with decreased risk of aGvHD and cGvHD; however, the probability of relapse and NRM were significantly increased [45]. Mossallam et al. [90] reported a statistical trend for reduced relapse risk with the presence of HLA‐E*01:03 in the patient or donor in another small cohort of identical sibling T‐cell replete transplants [90]. More recently, in a large cohort of 1629 haploidentical transplants, Petersdorf et al. [94] described protective associations of the patient genotype HLA‐E*01:03, 01:03 on NRM and mortality. Furthermore, they were able to show a link between donor receptor (NKG2A and NKG2C) and patient ligand (HLA‐E) genotype pairings, suggesting the precise combinations of these receptor‐ligand pairs are important in HCT patient clinical outcomes [94].

The data from these studies suggest a protective effect of the HLA‐E*01:03, 01:03 genotype on several of the common post‐transplant complications that can occur (Table 1). However, the impact of small and diverse cohort sizes may affect the data reported and contribute to the inconsistent findings observed. Many of these studies included patients and donors with matching *HLA‐E* genotypes; therefore, whether these effects originate from the patient or donor genotype is unclear and needs to be investigated explicitly in MUD transplants and in larger cohorts before conclusions can be drawn.

Several hypotheses for why HLA‐E*01:03 might be beneficial in the HCT setting have been proposed. Due to the known higher surface expression of HLA‐E*01:03 molecules, it is postulated that they are more effective at presenting pathogen‐derived peptides to CD8^+^ T cells, thus improving their ability to clear infections [84]. Higher expression levels of HLA‐E*01:03 are also thought to confer protection against the risk of GvHD due to the increased inhibition of NK cells via NKG2A, reducing their cytotoxicity against host cells [42, 47]. This effect could be especially pronounced in endothelial tissues as these cells have considerable HLA‐E expression [42, 43, 97]. Higher expression and peptide binding affinity of HLA‐E*01:03 were also proposed to be the cause of a reduced probability of relapse, due to the increased efficiency of peptide presentation to CD8^+^ T cells inducing a stronger graft‐versus‐leukaemia (GvL) effect [90, 98]. Another possible mechanism is through the competition of HLA‐E*01:03 with classical HLA class I molecules for peptides that would otherwise induce CD8^+^ T cell‐mediated cytotoxicity. It is proposed that the binding of these peptides by HLA‐E molecules reduces the available pool of peptides for classical HLA molecules to bind. In this situation, it is suggested that HLA‐E would present these peptides poorly, thus not inducing effective T cell responses [47]. Such mechanisms remain hypotheses until they can be explicitly shown in the HCT setting. Furthermore, only three studies have been able to show a donor genotype effect in explicitly MUD cohorts (Table 1). These are the most informative findings, as donor HLA‐E genotype is a variable that could be selected for during the unrelated donor selection process. It is vital to fill this gap in knowledge if *HLA‐E* is to be used in donor selection in the future.

### 6.2 Detrimental Impact of HLA‐E*01:03

In striking contrast to the early reports of a beneficial impact of the presence of HLA‐E*01:03 in either patient or donor genotypes in smaller and somewhat heterogeneous cohorts, larger studies failed to confirm these observations, conversely reporting a detrimental effect of this genotype (Table 1). In a cohort of 509 10/10 HLA, MUD HCTs where 63% of patients received a T‐cell depleted transplant and 63% of pairs were *HLA‐E* matched, patients with the HLA‐E*01:03, 01:03 genotype were found to have an increased probability of NRM and decreased DFS and overall survival, compared to patients with other genotypes [91].

The detrimental impact of HLA‐E*01:03 on the probability of DFS was also found by the largest *HLA‐E* study to date published in 2019, consisting of 1840 10/10 HLA, MUD transplant pairs, of which 67.5% were *HLA‐E* matched [92]. In this study, they reported an increased probability of disease relapse and decreased risk of DFS when donors with HLA‐E*01:03, 01:03 genotype were used [92]. Similar findings were observed in a large cohort of single HLA mismatched unrelated donor transplants primarily investigating the role of the HLA‐B leader peptide on transplant outcomes [95]. Here, the patient genotype HLA‐E*01:03, 01:03 was associated with increased relapse and decreased DFS [95]. Likewise, a similar study of 903 single HLA‐B mismatched (9/10) unrelated donor transplants identified a detriment to DFS with the donor HLA‐E*01:03, 01:03 genotype and an increase in risk of grade II‐IV aGvHD with the donor genotype HLA‐E*01:01, 01:03 [96]. Due to the HLA mismatched setting of these two cohorts, consideration must be taken if comparing results to those from matched transplants. The association of HLA‐E*01:03 homozygous donors with increased risk of disease relapse was also reported in a cohort of HLA identical sibling HCT in Mardani et al. [46]. The large 2019 study also split their cohort by the use of T‐cell depletion in the transplant regimen and reported increased risks of NRM and overall survival when patients received a graft from a donor with the HLA‐E*01:03, 01:03 genotype in patients undergoing a T‐cell replete transplant compared to those receiving T‐cell deplete grafts [92]. These findings were substantiated in a study of 93 related and MUD transplants, where patients receiving a graft from a donor with the HLA‐E*01:03, 01:03 genotype were reported to have a three‐fold increased risk of death [44]. Together, these studies suggest the use of related or unrelated donors with HLA‐E*01:03 alleles in their genotype could be correlated with worse patient prognosis post‐HCT, in contrary to the previously mentioned results (Table 1).

It has been hypothesised that the same proposed mechanism behind improved patient outcomes associated with the presence of HLA‐E*01:03 may also be responsible for detrimental outcomes. With its higher surface expression, HLA‐E*01:03 inhibits NK cells via the NKG2A receptor more effectively than HLA‐E*01:01, in doing so reducing NK cell cytotoxicity against the tumour, resulting in increased relapse [99]. Alternatively, Tsamadou et al. [92] suggested HLA‐E*01:01 may in fact bind tumour peptides more effectively, therefore inducing anti‐leukaemia responses of CD8^+^ T cells. While Tsamadou et al. [92] observed this effect only held true in patients receiving T cell replete transplants, Kordelas et al. [44, 92] reported only transplants using ATG for T cell depletion had an impact of donor *HLA‐E* genotyping on patient outcomes. This suggests that the effect *HLA‐E* genotype has on HCT outcomes could be strongly linked to the specific conditions of the individual transplant. Therefore, to understand in which situations *HLA‐E* genotype can be beneficial to transplant outcomes, future studies should focus on using homogeneous cohorts to give the best chance of being able to validate the findings.

### 6.3 *HLA‐E* Allele Matching

In contrast to classical HLA locus matching for HCT, fewer studies have identified an impact of direct *HLA‐E* allele matching on patient outcome. *HLA‐E* allele mismatching was reported to be a risk factor for increased mortality in a cohort of 460 otherwise HLA‐mismatched unrelated donors [88]. In contrast to all other studies discussed, each patient in this study cohort was mismatched for at least one classical HLA locus, potentially changing the immune environment after transplantation. Conversely, in a cohort of 509 10/10 HLA MUD HCTs, *HLA‐E* mismatching was associated with reduced probability of NRM and increased probability of DFS and OS, possibly suggesting that the single‐locus mismatches may be correlated with the observations in the first study and that the true impact of *HLA‐E* allele mismatching may have been masked [91]. The protective impact of *HLA‐E* mismatching was reported to be much more pronounced in patients with advanced disease stages compared to early/intermediate, in this second study [91].

*HLA‐E* mismatching has also been correlated with the risk of GvHD and infection. In two published abstracts describing the findings of 100 MUD and related donor HCTs, patients receiving *HLA‐E* mismatched grafts were reported to have increased risks of aGvHD and reduced risk of viral infection in comparison to *HLA‐E* matched patients [86]. The detrimental impact of *HLA‐E* mismatching on aGvHD risk was also reported more recently (71% vs. 57% for *HLA‐E* mismatched and matched pairs respectively) [93]. Tsamadou et al. [91] similarly reported that *HLA‐E* mismatching was associated with reduced risk of bacterial infection post‐HCT (9.5% vs. 17.2%); however, conversely to other studies, they found an improvement to both aGvHD (7.7% vs. 12.3%) and cGvHD [91]. The authors identified that this reduction in cGvHD was significant in patients with advanced disease stages, but no effect was seen in early/intermediate stages [91]. In contrast to these studies, the large study by Tsamadou et al. [92] was not able to confirm that *HLA‐E* matching had any significant effect on any of the measured endpoints. In a cohort of HLA‐B mismatched transplants, a reduction in the risk of relapse was observed with HLA‐E mismatches by Petersdorf et al. [96], but only if the mismatch was in the host‐versus‐graft direction. There are fewer studies investigating *HLA‐E* matching compared to patient or donor *HLA‐E* genotype in HCT, and it is obvious that additional, larger, and more homogeneous studies are needed to properly assess the effect of *HLA‐E* matching.

The proposed explanations for *HLA‐E* mismatching impacting HCT outcomes are that different *HLA‐E* alleles are able to present different repertoires of non‐canonical peptides in stress conditions, and these are proposed to preferentially bind to CD8^+^ T cells instead of NKG2A receptors [100]. The presence of mismatched *HLA‐E* alleles means the repertoire of peptides that can be presented by HLA‐E is increased, making it more likely for HLA‐E: peptide complexes to activate CD8^+^ T cells [91]. Tsamadou et al. [91] suggested this is one possible cause for the reduction in infection and increase in DFS observed, whereby *HLA‐E* mismatching induces a stronger immune response from NK and CD8^+^ T cells.

Interestingly, among all studies that included cohorts of 10/10 HLA MUD transplants, an average of only two thirds of patients and donors had identical *HLA‐E* genotypes (calculated from data in Table 1). This shows that even when matching *HLA* at the five classical loci typically considered for HCT, the inclusion of *HLA‐E* genotyping data would enable different haplotype identification in at least a third of cases. This number would undoubtedly increase by using full‐gene *HLA‐E* genotyping instead of exon‐based sequencing or other typing techniques, by identifying additional polymorphisms that would reveal different haplotypes. Our data from a cohort of 876 UK MUD HCTs, genotyped for *HLA‐E* using full‐gene TGS, corroborates this observation, where 60% of pairs were matched for *HLA‐E* at the CDS level, but decreases to 51% when matching for the whole *HLA‐E* gene is considered [73].

Classical *HLA* genes are the most important genes in transplant outcomes; however, there are many other genes in the MHC that could affect HCT outcome. While not necessarily functionally relevant, matching for these additional genes could be a proxy for a haplotype matched donor, which has been shown to reduce GvHD in patients post‐HCT [101, 102]. *HLA‐E* is located roughly in the middle of a 1.2 M base (Mb) region between *HLA‐A* and *HLA‐C. This* provides a greater possibility for a recombination event to have occurred between the two genes. If this occurred between two haplotypes with a common *HLA‐A* genotype, this could create distinct haplotypes that may not be identified by routine *HLA* genotyping strategies but differ from the common haplotype upstream of *HLA‐A* to the end of the chromosome. This explains the rationale for including *HLA‐E* in donor matching, helping to distinguish haplotypes that were identical across the classical HLA genes, thereby moving closer to a haplotype match as demonstrated recently by Sayer et al. [103]. The advantages of matching patients and donors by resolving more of the MHC or to a higher resolution by using ultra‐high resolution HLA genotyping have been demonstrated [104, 105]. Both of these studies found a reduction in aGvHD, and one found an improvement in overall survival associated with 12/12 ultra‐high resolution HLA genotyping [104, 105]. Additionally, in our cohort of 876 UK MUD HCTs, 24.7% of pairs that were previously matched at 12/12 HLA loci had mismatched *HLA‐E* alleles, showing *HLA‐E* matching is even able to distinguish haplotypes in this 12/12 ultra‐high resolution HLA genotyping setting [73].

## 7 Functional Implications of *HLA‐E* Diversity

Beyond the formerly discussed mechanisms, *HLA‐E* genotypes influence NK cell function in part through their involvement in NK cell education. Specifically, HLA‐E molecules are central to one of two mechanisms of NK cell education and therefore differences in HLA‐E expression levels and peptide repertoire could change the education of these cells [98]. A well‐documented example of this is the HLA‐B −21 M/T dimorphism: Methionine (−21M) leader sequences enhance surface expression and promote effective education of NKG2A^+^ NK cells which have a more potent cytotoxic potential, whereas Threonine (−21T) leaders do not [106, 107]. Consequently, alongside HLA‐B −21M/T, differential expression of HLA‐E proteins (e.g., HLA‐E*01:01 and HLA‐E*01:03) and differential peptide repertoires could also modulate NK cell functionality via this route [106, 108]. This HLA‐E‐dependent axis has been linked to stem cell transplantation outcomes across multiple settings [95, 96, 109, 110]. Interestingly, peptides that induce high expression of HLA‐E do not necessarily confer the strongest binding to NKG2 receptors and peptide variation outside the canonical nonamer also impacts HLA‐E presentation, highlighting the nuances of this pathway [111]. Following CMV reactivation, an expansion of NKG2C^+^ and corresponding reduction of NKG2A^+^ NK cells along with activation of NKG2A^+^ NK cells has been demonstrated [112]. This further supports an adaptive role of HLA‐E in shaping NK cell functionality and thus potentially GvL or GvHD responses. NK education calibrates the potency of NK cells and is crucial for generating effective immune responses, which is especially important in the context of HCT and with HLA‐E being central to this.

HLA‐E also modulates CD8^+^ T cell responses against infections like CMV and HIV by presenting a more diverse repertoire of viral peptides via TAP‐independent pathways that bypass CMV mediated TAP inhibition [113, 114, 115]. CMV enhances HLA‐E expression by mimicking the canonical VL9 peptide, evading NK cells but exposing itself to HLA‐E‐restricted CD8^+^ T cell responses [114]. In a cancer setting, these T cells can also recognise tumour‐derived peptides, contributing to GvL effects, including through TCR‐independent mechanisms [116, 117]. Notably, unlike conventional CD8^+^ T cells, the selection by either thymic epithelial or haematopoietic cells shapes the phenotype and effector functions of HLA‐E‐restricted CD8^+^ T cells [118, 119].

There are several factors that make establishing a consensus on the impact of *HLA‐E* genotyping data on HCT outcomes difficult: the overall shortage of studies, small cohort sizes, heterogeneity in transplant conditions and limited *HLA‐E* genotyping (Table 1). Most cohorts are small and the reported data differences between them are likely indicative of this limitation. Two studies to date have included more than 500 transplant pairs within their study cohorts, which has led to more convincing findings, but that still require confirmation in independent, and possibly larger cohorts, before they could be considered for transplantation into clinical practice. Additionally, as shown in Table 1, the transplant protocols and conditions vary greatly between each study; therefore, the reported effects may not persist in other settings, which makes understanding the underlying biology difficult. Finally, all studies characterised *HLA‐E* alleles based on limited regions of the gene and in many cases using non‐sequencing techniques. Exon 3 was the only region genotyped across all studies and only three studies also included exon 2, likely because when most of these studies were published, few *HLA‐E* alleles had been identified and even less was known about intronic polymorphism within the gene. As such, what is being reported by one study as an HLA‐E*01:03, for example, might not be the same as an HLA‐E*01:03 in another study. All of these disparities between studies only muddies the water further and makes identifying the truth beneath more difficult.

To summarise, there are two leading avenues for investigating the impact of *HLA‐E* genotypes on HCT patient outcomes. Selecting donor *HLA‐E* alleles for their functional differences, as is presumed to be the case with HLA‐E*01:01 versus HLA‐E*01:03, or matching patient and donor *HLA‐E* genotypes for distinguishing otherwise identical haplotypes and reducing incompatibility across other regions of the MHC. Whether one or both of these avenues can be truly impactful on HCT outcomes is not yet known, especially when considering how the nuances of different transplant conditions could alter the relationship of HLA‐E with patient outcomes. Evidently, the role of *HLA‐E* genotypes and matching in HCT remains unclear; however, with additional retrospective studies on larger cohorts, a clearer picture into the impact of *HLA‐E* genotype may become evident.

## Conflicts of Interest

The authors declare no conflicts of interest.

## Data Availability

The data that support the findings of this study are available from the corresponding author upon reasonable request.
